# Antibiotics for the Secondary Prevention of Coronary Heart Disease

**DOI:** 10.12968/bjca.2022.0082

**Published:** 2022-10-02

**Authors:** M. Mansoor, O Hamer, E Walker, J Hill

**Keywords:** Antibiotics, Coronary heart disease, Systematic review, Randomised control trial, Critical appraisal, Drug therapy

## Abstract

It is estimated that 200 million people are living with coronary heart disease, which remains one of the leading causes of mortality and morbidity worldwide. Those living with coronary heart disease are at an increased risk of cardiovascular events such as stroke, myocardial infarction, and cardiovascular death. Pathophysiology of coronary heart disease revolves around inflammation which leads to plaque build-up. Antibiotics are known to hold anti-inflammatory and anti-oxidative properties. It is theorized that reductions in inflammation could prevent cardiovascular events which may reduce suffering, risk of death and hospital admission rates in patients with coronary heart disease. This article critically appraises a systematic review that assessed the risk of antibiotics used as secondary prevention for coronary heart disease.

## Introduction

With a substantial number of deaths caused by coronary heart disease (CHD) each year, it remains the leading cause of mortality and morbidity worldwide (1). It is estimated that 200 million people around the world are living with coronary heart disease (1). Increased prevalence is exacerbated by the growing frequency of obesity, diabetes, and an ageing population (1). Coronary heart disease is a condition in which the heart’s blood supply is stopped or interrupted, commonly through a build-up of lipid deposition and plaque formation in the coronary arteries, known as atherosclerosis (1, 2). The interrupted flow of blood to the heart often results in a restriction of oxygen (3). Over some years, repeated restriction of oxygen weakens the heart muscle and increases the risk of myocardial infarction (3). Typical symptoms of coronary heart disease include jaw pain, arm pain, and chest pain (described as tight and crushing) which is often intensified with physical activity (4).

Those living with coronary heart disease are known to be at an increased risk of cardiovascular events such as stroke, limb ischaemia and cardiovascular death (5). These events contribute to poor quality of life, mortality, and health inequalities (6). Adjunctive drug therapies (including antihypertensives, statins or anti-thrombotics) and lifestyle interventions (such as weight reduction and smoking cessation), aim to reduce the risk of cardiovascular events (7). Consequently, these interventions help to reduce suffering, risk of death and hospital admission rates (7). However, even total adherence to these treatments is not enough to eliminate cardiovascular risk (7).

A significant proportion of people with coronary heart disease receive antibiotics each year (8). Antibiotics such as macrolides are commonly used to treat and prevent bacterial infections (through inhibition or cessation of bacterial growth), and are known to hold anti-inflammatory and anti-oxidative properties (9). There is evidence to suggest that inflammation, possibly caused by infectious agents, is associated with plaque formation in atherosclerosis, which causes and exacerbates coronary heart disease (10). Some evidence suggests that antibiotics can reduce the inflammation that exacerbates plaque formation (a leading cause of coronary heart disease), contributing to the prevention of mortality and morbidity from coronary heart disease (11). However, there is a dearth of high-quality evidence to support antibiotic use as a secondary prevention method of coronary heart disease (12). Notably, existing studies that have investigated the association (antibiotics and coronary heart disease), were conducted prior to 2009 and reported inconsistent findings related to outcomes of morbidity and mortality (13–15). Given the diversity of findings, a meta synthesis was needed to provide guidance to clinical practise and policy on how antibiotics should be employed in relation to the secondary prevention of coronary heart disease (12). The Cochrane review by Sethi et al, synthesised the evidence relating to the use of antibiotic in secondary prevention of coronary heart disease (12).

### Aim of commentary

This commentary aims to critically appraise the methods used within the review by Sethi et al and expand upon the findings in the context of clinical practice (12).

### Methods of the systematic review by Sethi et al, 2021

The Cochrane systematic review employed a comprehensive search strategy including six databases from inception to December 2019: Cochrane Central Register of Controlled Trials (CENTRAL), MEDLINE (Ovid), Embase (Ovid), BIOSIS (Web of Science), SCI-Expanded (Web of Science) and LILACS (Bireme). The review also searched 10 trials registries, The Turning Research into Practice (TRIP) Database and Google Scholar for ongoing trials. No restrictions related to language or publication type were imposed on the search strategies. Further to the searches, reference lists of included studies were screened for any unidentified trials (12).

The review employed a robust criterion for the selection of studies. Trials were included if they assessed the beneficial or harmful effects of antibiotics (compared to placebo or no intervention) for the secondary prevention of coronary heart disease, in adults (≥ 18 years) with a diagnosis of coronary heart disease. All types of antibiotics were included irrespective of dose, duration, or route of administration. Trials were included irrespective of setting, blinding, publication status, publication year, language, and reporting of outcomes. Participants were included irrespective of sex and antibody status (e.g., for C pneumoniae, H pylori, P gingivalis, or E coli). Trials were excluded if they included participants with chronic inflammatory diseases, or if they compared antibiotics with active pharmaceutical medication (12).

Screening of titles and abstracts was conducted independently by two review authors. Full text reports were screened independently by three review authors, with any disagreements resolved by a fourth author. Data extraction was undertaken independently by three review authors, with any disagreements resolved by a fourth review author. The Cochrane Risk of Bias tool was independently used by three review authors to critically appraise included trials (16). An assessment to establish the certainty of evidence for each outcome (rating of certainty) was conducted using the Grading of Recommendations Assessment, Development and Evaluation (GRADE) system (17).

The review comprehensively outlined the outcomes and when the measurement of the outcomes took place. Outcome measures were extracted at two time points: maximum follow-up (the time point of primary interest) and 24±6 months follow-up. Three primary outcomes were extracted which included all-cause mortality, serious adverse events, and quality of life. The secondary and post hoc outcomes of interest were cardiovascular mortality, myocardial infarction, stroke, sudden cardiac death, hospitalisation for any cause, revascularisation, and unstable angina pectoris. Data synthesis was undertaken using both random-effects and fixed effects model meta-analysis calculating risk ratios, absolute risk reduction and number needed to treat for an additional beneficial when combining outcomes across multiple studies.

### Results of the systematic review by Sethi et al, 2021

The Cochrane systematic review included 61 publications reporting on a total of 38 trials. These trials compared antibiotics versus placebo or no intervention in patients with CHD totalling 26,638 participants (12). The trials were conducted from 27 different countries. The mean age was 61.6 years. The mean proportion of women was 22.9%. Participants had the following conditions: 25.6% currently smoked, 19.6% had diabetes, 51% had hypertension and 66.2% had hyperlipidaemia.

### Primary outcome 1 -All-cause mortality

Meta-analysis using random effects model showed a slight increase in risk of all-cause mortality within groups who received antibiotics versus placebo or no intervention at a maximum follow up of 3-120 months (RR 1.06, 95% CI 0.99 to 1.13, P=0.07, GRADE: High certainty). At 24 +/- 6 months follow up there was a slight increased risk of all-cause mortality in groups who received antibiotics versus placebo or no intervention (RR 1.25, 95% CI 1.06 to 1.48, P = 0.007, GRADE: High certainty).

### Primary outcome 2 – Serious adverse events

None of the included trials reported serious adverse events according to the definition within the inclusion criteria, either at maximum follow-up or at 24±6 months follow-up.

### Primary outcome 3 – Quality of life

None of the included trials reported any data on quality of life either at maximum follow-up or at 24±6 months follow-up.

### Secondary outcome 1 - Cardiovascular mortality

Meta-analysis using a random effects model showed a non-statistically significant slight increase in risk of cardiovascular mortality within groups who received antibiotics versus placebo or no intervention at maximum follow up 24 -120 months (RR 1.11, 95% CI 0.98-1.25, P= 0.11, GRADE: Moderate certainty).

At 24 +/- 6 months follow-up there was a statistically significant increased risk of cardiovascular mortality within groups who received antibiotics versus placebo or no intervention (RR 1.5, 95% CI 1.17 to 1.91, P=0.001, GRADE: High certainty)

### Secondary outcome 2 – Myocardial infarction

There was no evidence of difference in risk of myocardial infarction within groups who received antibiotics versus placebo or no intervention at a maximum follow-up of 3-120 months (GRADE: High certainty), and 24 +/- 6 months (GRADE: Moderate certainty).

### Secondary outcome 3 - Stroke

Meta-analysis using fixed effect models showed a significant increase in risk of stroke within groups who received antibiotics versus placebo or no intervention at a maximum follow up 6-120 months (RR 1.14, 95% CI 1.00 to 1.29, P=0.04, GRADE: High certainty). At 24 +/- 6 months there was no evidence of difference in risk of stroke within groups who received antibiotics versus placebo or no intervention (GRADE: High certainty).

### Secondary outcome 4 – Sudden cardiac death

There was no evidence of difference in risk of sudden death within groups who received antibiotics versus placebo or no intervention at a maximal follow up of 18.5 -120 months (GRADE: Moderate certainty). At 24 +/- 6 months there was a statistically significant increase in risk of sudden death within groups who received antibiotics versus placebo or no intervention (RR 1.77, 95% CI 1.28 to 2.44, P=0.0005, GRADE: Moderate certainty).

### Additional post hoc outcomes

Three additional post hoc outcomes were analysed: hospitalisation for any cause, revascularisation, and unstable angina pectoris. All three outcomes were analysed using a fixed effects model showing no evidence of difference in risk between groups who received antibiotics versus placebo or no intervention (P= >0.05). The evidence relating to the three post hoc outcomes was graded at moderate to high certainty with little to no indication of any heterogeneity.

### Commentary on the review by Sethi et al, 2021

Using the AMSTAR 2 critical appraisal tool for systematic reviews, 14 out of the 16 criteria were judged to be of satisfactory for this review (18). Two criteria were not met: The literature search strategy did not include grey literature, and the sources of funding for included studies were not consistently detailed in the review (18). This said, concerns regarding a lack of grey literature searches may not be important given the inclusion criteria to only include trials. Overall, the systematic review was judged to provide an accurate and comprehensive summary of the results of the available studies that address the question of interest (12). Based upon this appraisal it was deemed that the systematic review provides accurate and comprehensive synthesis of the available studies that address the question of interest.

Within clinical practice, antibiotics are generally used to fight active infections through their ability to kill and/or inhibit growth of bacteria (19). Studies have theorized that antibiotics have immunomodulatory anti-inflammatory effects and could decrease the risk of coronary heart disease (and its complications) (9). However, this review found that antibiotic use as secondary prevention, may slightly increase the risk of all-cause mortality and stroke in adults (18 years plus) with CHD. These findings were based upon high certainty of evidence (GRADE) which means the authors believe that the estimated effect is very close to the true effect (12, 20, 21). The review also established that antibiotic use for secondary prevention of CHD may significantly increase the risk of cardiovascular mortality and sudden cardiac death (18 years plus) (12). These conclusions were based upon moderate to high certainty of evidence (GRADE) which means the authors believe that the estimated effect is near to the true effect (12, 21).

The review concludes with moderate to high certainty that patients who take antibiotics for the purpose of secondary prevention of CHD are at a significantly increased risk of harm by death or stroke (compared to CHD patients who received placebo or no intervention) (12). Although the mechanism is unclear, there are numerous explanations that have been proposed by various research papers (22, 23). Firstly, findings from Nguyen et al, suggest that some antibiotics exacerbate inflammation rather than act to reduce inflammation (24). There is a rationale to suggest that inflammation could be a concern, given that research has identified higher cumulative broad spectrum antibiotic use is associated with increased risk of developing inflammatory bowel disease (a risk factor for developing cardiovascular disease) (23, 25). Secondly, antibiotics are known immunomodulators of the immune system and as a consequence may induce a broad range of pro-inflammatory effects (alongside anti-inflammatory effects) (26). A further explanation is that specific classes of antibiotics (e.g., macrolides, quinolones and tetracyclines) can prolong heart rate contraction to relaxation intervals, often causing arrythmias which increase the risk of cardiac arrest and stroke (via their effect on potassium channels) (27, 28). This being said, adverse events are mainly reported in patients with pre-existing conditions who have been administered with macrolides (27). Lastly, some research has suggested that the use of antibiotic greater than two months may induce alterations in microbiota composition (even after cessation of the treatment), which could increase platelet hyperreactivity and propensity to thrombosis (22). For clinical practice, these contraindications mean that healthcare professionals need to carefully consider antibiotic use, especially macrolides, quinolones and tetracyclines for the purpose of secondary prevention in adults with CHD, as they may cause harm to patients. The existence evidence does not provide findings to establish the benefits or harms of other antibiotic classes (e.g., sulfonamides, b-lactams) for secondary prevention of CHD.

Future research should focus on examining a larger subset of antibiotics as only macrolides, quinolones and tetracyclines were included in the review. However, given the finding of the review it may not be ethical to conduct further clinical trials (12). Instead, retrospective observational studies should be conducted to examine the beneficial and harmful effects of these other antibiotics for the secondary prevention of CHD. Explicitly, antibiotics such as penicillin’s and cephalosporins require further research so that clinical guidance about their use for the secondary prevention of coronary heart disease can be provided. Future research should also focus on coronary heart disease with an active infection and antibiotics as there is a dearth of literature in this area. Finally, research should focus on antibiotic use in late stages coronary heart disease but before events such as myocardial infarction has occurred, to observe if there is any benefit of administration.

## Key Points

1. When compared to placebo or no intervention, antibiotics may increase the risk of all-cause mortality, cardiovascular mortality, and sudden cardiac death at 24 +/- 6 months follow-up in patients with coronary heart disease.
2. When compared to placebo or no intervention, antibiotics may slightly increase the risk of stroke at maximum follow up (6 -120 months) in patients with coronary heart disease.
3. Clinicians should carefully consider and perform a risk/benefit analysis before giving antibiotics for secondary prevention of coronary heart disease.
4. Future retrospective observational research should focus on expanding the types of antibiotics included and conduct research on specific sub-groups of patients with early, mid, and late stages of coronary heart disease.

## CPD reflective questions

1. What are the limitations and strengths of the systematic reviews?
2. Why is the GRADE approach in systematic reviews used and what are its benefits?
3. What are the practical considerations when prescribing antibiotics?
